# Complement system in kidney transplantation: from innate immune surveillance to a therapeutic and genetic engineering target

**DOI:** 10.3389/fimmu.2026.1961041

**Published:** 2026-09-28

**Authors:** Yoichi Kakuta, Shuji Miyagawa, Shunsuke Mori, Soichi Matsumura, Masataka Kawamura, Shigeaki Nakazawa, Akira Maeda, Shunsuke Saito, Masahito Ikawa, Miho Watanabe, Shigeru Miyagawa, Norio Nonomura

**Affiliations:** 1 Department of Urology, The University of Osaka Graduate School of Medicine, Suita, Osaka, Japan; 2 Department of Pediatric Surgery, The University of Osaka Graduate School of Medicine, Suita, Osaka, Japan; 3 Department of Cardiovascular Surgery, The University of Osaka Graduate School of Medicine, Suita, Osaka, Japan; 4 Department of Experimental Genome Research, Research Institute for Microbial Diseases, The University of Osaka Graduate School of Medicine, Suita, Osaka, Japan

**Keywords:** antibody-mediated rejection, complement system, innate immunity, kidney allograft, kidney transplantation, kidney xenotransplantation

## Abstract

Kidney transplantation remains the most effective treatment for end-stage kidney disease; however, long-term graft survival is constrained by rejection and chronic graft injury, and donor shortages continue to drive interest in xenotransplantation. The complement system is increasingly recognized both as a terminal effector of antibody-mediated injury and as a regulatory network linking antibody responses, endothelial activation, ischemia–reperfusion injury, coagulation, and innate immune cell responses. In this review, we compare the role of complement in allogeneic and xenogeneic kidney transplantation and provide an overview of emerging therapeutic strategies. In allogeneic transplantation, donor-specific anti-HLA and anti-ABO blood group antibodies activate complement, particularly via the classical pathway, and contribute to antibody-mediated rejection. Although C4d deposition is a key marker of complement activation, its clinical significance depends on the presence of microvascular inflammation, endothelial injury, and other immune mechanisms. Complement-binding donor-specific antibodies may improve risk stratification, whereas the clinical benefits of inhibiting C5, C3, and upstream complement in chronic active antibody-mediated rejection remain uncertain. In xenotransplantation, α-Gal and non-Gal antigens, their corresponding natural antibodies, complement activation via the classical, lectin, and alternative pathways, and interspecies incompatibilities in complement regulation collectively promote vascular endothelial injury and thromboinflammation. Multiplex genetic modification of donor pigs, including deletion of major carbohydrate antigens and expression of human complement and coagulation regulatory proteins, has substantially mitigated hyperacute rejection. However, achieving durable xenograft survival requires effective control of macrophages, natural killer cells, neutrophils, and their interactions with the graft endothelium. Therefore, integrated strategies combining genetic complement regulation, pharmacological complement inhibition, coagulation regulation, and innate immune modulation may be essential for preventing chronic allograft injury and improving long-term kidney xenograft survival.

## 1 Introduction

Although kidney transplantation remains the most effective treatment for end-stage kidney disease, long-term outcomes are compromised by rejection. Moreover, the global shortage of organ donors continues to be a major unresolved challenge (1–3). Traditionally, the complement system has been identified as a major effector of hyperacute and antibody-mediated rejection (ABMR). However, emerging research indicates that complement functions not only as a terminal effector of antibody-mediated tissue injury but rather as a network that regulates inflammatory responses within the graft through multiple mechanisms. Therefore, the complement system serves as a key regulatory mechanism that amplifies inflammatory reactions induced by antibody-mediated endothelial injury and links them to coagulation abnormalities, ischemia–reperfusion injury, and activation of innate immune cells, thus contributing to chronic graft injury (4, 5). These diverse functions of complement are particularly evident when comparing allogeneic kidney transplantation with xenogeneic kidney transplantation (6).

In allogeneic kidney transplantation, complement activation triggered by donor-specific anti-HLA antibodies (DSA) and anti-ABO blood group antibodies play a key role in the pathogenesis of ABMR (7, 8). Desensitization enables selected patients with preformed anti-HLA antibodies to undergo kidney transplantation and is associated with a survival benefit (9, 10). However, no effective treatment strategies have been established for chronic active ABMR. Therefore, elucidating the mechanisms that perpetuate inflammation and endothelial injury, including complement activation, is essential for improving long-term kidney allograft survival (11, 12).

Robust natural antibody responses and complement activation present even more significant challenges in xenogeneic kidney transplantation than in allogeneic transplantation (13). Recently, genetically modified pigs have been developed that are characterized by the expression of human complement regulatory proteins, coagulation regulatory factors, innate immune regulatory molecules, and knockout of key genes associated with xenoantigens. These advances have markedly reduced hyperacute rejection and enabled short-term kidney graft function in limited human studies (14, 15). However, clinically reproducible long-term graft survival has not yet been achieved. Thus, strategies combining the effective modulation of complement and innate immune responses, including neutrophils and macrophages, are likely to be essential for successful long-term xenogeneic kidney transplantation (13, 16).

In this review, we focus primarily on kidney transplantation and compare the roles of the complement system in allogeneic and xenogeneic transplantation. Rather than considering complement solely as a defense mechanism in innate immunity, we explore its role as a therapeutic target for improving transplant outcomes. Furthermore, we discuss how complement activation contributes to endothelial injury, coagulation abnormalities, ischemia–reperfusion injury, and innate immune cell responses; highlight its pathophysiological significance; and outline future therapeutic strategies.

## 2 Complement activation and regulation in transplantation

The complement system is a central humoral component of innate immunity that plays a key role in pathogen and foreign substance recognition, inflammation amplification, cell injury, and clearance of immune complexes. Complement activation is initiated mainly through three pathways: the classical, lectin, and alternative pathways. The classical pathway is triggered by antigen–antibody complexes, whereas the lectin pathway is activated when mannose-binding lectin or ficolins recognize carbohydrate structures on target surfaces. Conversely, the alternative pathway is activated via spontaneous C3 hydrolysis or C3b deposition on target surfaces. The alternative pathway amplifies complement activation initiated through the classical and lectin pathways. Granzyme K activates complement through a noncanonical mechanism involving the direct cleavage of C2 and C4. However, its relevance to transplantation remains to be established (17). All three pathways converge at C3 activation and collectively induce inflammatory responses and cell injury through C3a, C3b, C5a, and the C5b-9 membrane attack complex (MAC) (4, 5, 18).

In transplantation, complement activation does not merely serve as a mechanism for eliminating foreign substances but rather as a key driver of graft injury (**Figure 1**). The binding of antibodies to graft vascular endothelium activates the classical pathway. Damage-associated molecular patterns (DAMPs) released during ischemia–reperfusion injury or tissue damage may promote complement activation through the lectin and alternative pathways. In addition, C3b deposited on the graft surface amplifies complement reactions through the alternative pathway and drives local inflammation. C3a and C5a recruit and activate leukocytes such as neutrophils, monocytes, and macrophages and promote the expression of adhesion molecules and inflammatory cytokines by endothelial cells. The MAC not only directly injures the cell membranes of target cells but also activates endothelial cells at sublytic levels, thereby exacerbating inflammation, coagulation, and platelet activation (19–21).

**Figure 1:**
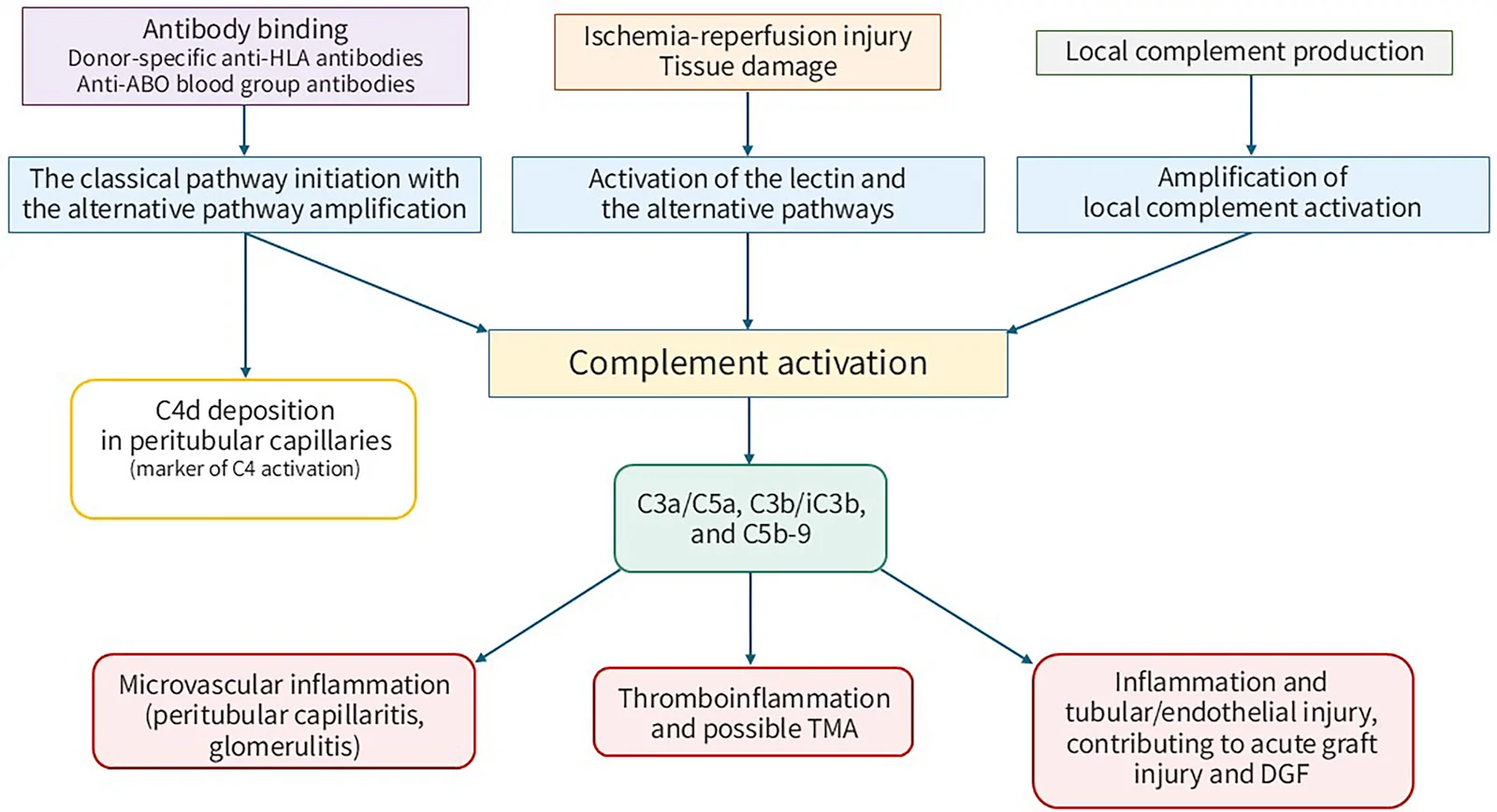
Complement-mediated graft injury in kidney allotransplantation. Binding of donor-specific anti-HLA or anti-ABO blood group antibodies to graft endothelium initiates complement activation primarily through the classical pathway, with amplification through the alternative pathway. Ischemia–reperfusion injury and tissue damage activate the lectin and alternative pathways, whereas local complement production by tubular epithelial cells amplifies complement activation. Complement effectors, including C3a/C5a, C3b/iC3b, and C5b-9, promote microvascular inflammation, thromboinflammation and possible thrombotic microangiopathy, and tubular and endothelial injury. C4d deposition in peritubular capillaries is a marker of C4 activation and antibody–tissue interaction. However, C4d positivity alone does not establish antibody-mediated rejection and may reflect accommodation in ABO-incompatible transplantation. Tubular and endothelial injury may contribute to acute graft injury and delayed graft function. DGF, delayed graft function; TMA, thrombotic microangiopathy; HLA, human leukocyte antigen.

Living organisms have developed complement regulatory mechanisms to protect their tissues from excessive complement activation. The key membrane-bound complement regulatory proteins include CD46 (membrane cofactor protein, MCP), CD55 (decay-accelerating factor, DAF), and CD59. CD46 functions as a cofactor for factor I to promote the inactivation of C3b and C4b. CD55 accelerates the decay of C3/C5 convertases. CD59 acts on the terminal complement pathway and suppresses MAC formation. Because these proteins regulate distinct stages of the complement cascade, no single regulatory mechanism can effectively suppress the entire complement activation process (22).

Collectively, this demonstrates that, in transplantation, the complement system functions as a multilayered regulatory network that integrates inflammation, coagulation, endothelial activation, and innate immune cell responses. In allogeneic kidney transplantation, the classical pathway activation mediated by DSA or anti-ABO antibodies plays a key role, particularly in ABMR. Conversely, in xenogeneic kidney transplantation, complement activation is driven in a complex manner by natural antibodies, non-Gal antigens, the lectin pathway, amplification via the alternative pathway, and interspecies differences in complement regulatory proteins. Therefore, therapeutic targeting of complement should consider the specific pathway to be regulated, the timing of intervention, and the site of intervention according to the underlying pathology.

## 3 Complement in allogeneic kidney transplantation

In allogeneic kidney transplantation, the role of the complement system is most clearly demonstrated during ABMR. In recipients with DSA, antibodies bind to HLA antigens on the vascular endothelium of the kidney allograft and activate the complement cascade primarily through the classical pathway. During this process, C4d is deposited in peritubular capillaries, which has long served as a histological marker of complement activation in the diagnosis of ABMR (7, 23).

Complement activation amplifies local inflammatory responses within the graft. C3a and C5a, generated by the cleavage of C3 and C5, recruit and activate leukocytes such as neutrophils, monocytes, and macrophages; promote endothelial activation; and enhance vascular permeability. Moreover, the deposition of C3b and iC3b on the surface of antibody-coated target cells enhances interactions with effector cells such as NK cells, macrophages, and neutrophils through complement receptors. These interactions amplify antibody-dependent cellular cytotoxicity and phagocytic responses. Pathologically, these reactions manifest as microvascular inflammation (MVI), including glomerulitis and peritubular capillaritis, which are characteristic features of graft injury in ABMR (7, 23–25). Therefore, ABMR is not merely a consequence of antibody deposition or complement activation alone but rather is a complement-dependent inflammatory reaction targeting the microvascular endothelium of the kidney allograft.

In ABO-incompatible kidney transplantation, anti-ABO blood group antibodies induce complement activation by binding to blood group antigens expressed on the vascular endothelium. However, unlike in anti-HLA antibody-mediated ABMR, C4d deposition in peritubular capillaries is frequently observed in ABO-incompatible kidney transplantation, without accompanying overt tissue injury (8, 26, 27). In cases with C4d deposition without MVI, such as glomerulitis or peritubular capillaritis, C4d deposition itself may have a limited impact on graft prognosis (28). This indicates that C4d should be interpreted not only as a direct indicator of complement activation but rather in combination with the presence or absence of MVI and endothelial injury.

Although the presence of DSAs is a key risk factor for ABMR and graft loss, not all DSAs exhibit the same pathogenicity. Recent studies have indicated that complement-binding DSAs, such as C1q- and C3d-binding DSAs, are associated with a greater risk of graft loss (29, 30). This suggests that assessing complement-activating capacity, as well as the presence or titer of DSA, may be useful for risk stratification in ABMR. Furthermore, donor–recipient HLA differences can be assessed at the eplet level, with eplets representing small configurations of polymorphic amino acid residues on the HLA molecular surface. Eplet mismatch, particularly involving HLA-DQ, is associated with *de novo* DSA formation, rejection, and graft failure and may provide a more refined assessment of alloimmune risk than conventional HLA antigen mismatch counts (31, 32). However, the existence of C4d-negative ABMR and complement-independent endothelial injury indicates that, although complement activation plays a key role in ABMR, it does not fully encompass the entire spectrum of ABMR (23, 25, 33). More recently, killer-cell immunoglobulin-like receptor (KIR) ligand mismatch, specifically NK cell activation via missing-self recognition, has been implicated in MVI and ABMR-like endothelial injury (34). This suggests that microvascular injury in kidney allografts is not driven solely by antibodies and complement but is also influenced by innate immune responses involving NK cells.

Current therapeutic approaches for ABMR aim to reduce circulating alloantibodies and suppress their continued production. Either plasma exchange or immunoadsorption, often combined with intravenous immunoglobulin, is used to remove or neutralize circulating antibodies, whereas B cell- and plasma cell-directed therapies have been investigated to limit ongoing antibody production. However, particularly in the case of chronic active ABMR, the efficacy of these interventions remains inconsistent, and standardized treatment has yet to be established (11, 12).

Against this background, complement-targeted therapies have been developed to inhibit the downstream effector mechanisms of antibody-mediated injury, including C5 inhibitors, C1 esterase inhibitor (C1-INH), and C3 inhibitors. Eculizumab, an anti-C5 antibody, inhibits C5 cleavage, thereby suppressing the production of C5a and MAC formation. Although some studies have reported a reduction in early ABMR with eculizumab in sensitized kidney transplant recipients, its effects on long-term graft survival and prevention of chronic active ABMR remain uncertain (35, 36). Moreover, whether complement inhibition alone can sufficiently improve long-term outcomes remains unclear (7, 11, 36). C1-INH has also been evaluated as a therapy targeting more upstream stages of complement activation; however, further validation is warranted to establish its clinical utility (37).

In addition to ABMR, complement may also be involved in T cell-mediated immune responses. Biopsy specimens with T cell-mediated rejection (TCMR) have demonstrated increased expression of complement-related genes, including *C3*, *CFB*, *CFH*, and *C1S* (38). Furthermore, the tubular epithelial cells in the kidney allograft may locally synthesize complement components and modulate T cell responses and interstitial inflammation (39). In addition to complement activation, the disruption of complement regulation plays a key pathogenic role in graft tissue. We previously reported that in rat and human kidney grafts the expression of complement regulatory factors, including CD46/MCP, CD55/DAF, and CD59, decreases with the progression of acute TCMR (40). This suggests that graft injury in TCMR is driven not only by cellular immune reactions but also by local complement activation and reduced complement regulatory capacity. Therefore, in allogeneic kidney transplantation, complement should be regarded both as a mechanism of the classical pathway activation in ABMR as well as a pathogenic factor linking ischemia–reperfusion injury, local inflammation, TCMR, and chronic graft injury.

Taken together, these observations indicate that the complement system plays multiple roles in allogeneic kidney transplantation. This system mediates graft injury, provides diagnostic and risk-stratification biomarkers, and represents a potential therapeutic target. Future studies should aim to define the patients and pathological contexts in which complement is a dominant driver of injury and determine how complement inhibition can be optimally integrated with antibody removal and therapies targeting B cells and plasma cells.

## 4 Complement in xenogeneic kidney transplantation

Compared with allogeneic kidney transplantation, xenogeneic kidney transplantation is characterized by stronger natural antibody responses and complement activation, which represent the central mechanisms of graft injury. In pig-to-human xenotransplantation, natural human antibodies recognize xenogeneic antigens on porcine vascular endothelium and rapidly activate the complement cascade primarily through the classical pathway. This reaction is accompanied by endothelial injury, platelet activation, and thrombosis, which are collectively major contributors to hyperacute rejection in unmodified porcine organs (6, 13).

Major xenogeneic antigens include α-Gal, Neu5Gc, and the Sd(a) antigen. α-Gal is a carbohydrate antigen synthesized by GGTA1 and is absent in humans and Old-World monkeys; thus, anti-α-Gal antibodies are naturally present in human blood. Similarly, Neu5Gc, a non-Gal antigen associated with CMAH, is not synthesized in humans, making it a potential target of anti-Neu5Gc antibodies. The Sd(a) antigen, synthesized by β-1,4-N-acetylgalactosaminyltransferase 2 encoded by B4GALNT2, may also be involved in residual antibody responses and complement activation. Consequently, current genetically modified pigs feature a triple knockout of *GGTA1*, *CMAH*, and *B4GALNT2* to suppress antibody- and complement-mediated injury (41, 42). Additionally, targeting both B4GALNT2 and B4GALNT2-like may be necessary for more complete suppression of Sd(a)-related antigen expression (43).

Elimination of major xenogeneic antigens does not completely prevent complement activation. Residual antibodies against non-Gal antigens, DAMPs associated with ischemia–reperfusion injury, endothelial activation, and amplification through the lectin and alternative pathways overlap, resulting in the involvement of multiple complement pathways within the graft. In particular, the alternative pathway enhances C3b deposition initiated through the classical or lectin pathways and sustains complement reactions. Moreover, porcine complement regulatory proteins do not adequately regulate human complement, and these intrinsic interspecies differences promote complement-mediated vascular injury (6, 13, 21, 22).

Recent genetically engineered donor pigs have evolved from single-gene knockout models to more complex multiplex-edited pigs that combine triple knockout of major carbohydrate antigens, expression of human complement and coagulation regulatory transgenes, innate immune regulatory molecules, and, in certain platforms, inactivation of porcine endogenous retroviruses. These advances have markedly reduced hyperacute rejection and enabled short-term kidney graft function in nonhuman primate models and limited human studies (14, 15, 44). The major genetic modifications implemented in donor pigs are summarized in **Table 1**.

**Table 1:** Genetic modifications implemented in donor pigs for xenogeneic kidney transplantation.

Category	Genetic modification	Primary purpose	References
Major carbohydrate antigen deletion	*GGTA1* KO, *CMAH* KO, *B4GALNT2* KO	Eliminates α-Gal, Neu5Gc, and Sd(a) antigens to suppress antibody binding and subsequent complement activation	(41, 42, 44)
Complement regulation	hCD46/MCP, hCD55/DAF, and hCD59 in earlier transgenic pig lines	Promotes C3b/C4b inactivation, accelerates C3/C5 convertases decay, and inhibits terminal complement-mediated injury	(22, 44, 45)
Coagulation regulation	Human thrombomodulin/THBD, human EPCR/PROCR	Enhances compatibility with human protein C pathway and reduces coagulation dysregulation and thrombotic microangiopathy	(44, 46–48)
Innate immune regulation	hCD47	Provides a “don’t eat me” signal that suppresses macrophage-mediated phagocytosis and cytotoxicity	(44, 49)
Anti-inflammatory/cytoprotective regulation	HMOX1, TNFAIP3/A20	Attenuates endothelial inflammation, apoptosis, and ischemia–reperfusion-associated injury	(44)
Viral risk reduction	PERV inactivation	Reduces potential risk associated with porcine endogenous retroviruses; not directly related to complement-mediated injury	(44)
Multiplex donor design	Combination of 3KO, human complement regulatory genes, coagulation regulatory genes, innate immune/cytoprotective genes, and, in some platforms, PERV inactivation	Provides multilayered regulation of antibody binding, complement activation, endothelial activation, coagulation dysregulation, and innate immune injury	(14, 15, 44)

3KO, triple knockout; DAF, decay-accelerating factor; EPCR, endothelial protein C receptor; HMOX1, heme oxygenase 1; KO, knockout; MCP, membrane cofactor protein; PERV, porcine endogenous retrovirus; PROCR, protein C receptor; THBD, thrombomodulin; TNFAIP3/A20, tumor necrosis factor alpha-induced protein 3.

However, after avoiding hyperacute rejection, durable xenograft survival remains limited by several interconnected immunological and vascular barriers. Residual preformed and *de novo* xenoreactive antibodies against non-Gal and SLA antigens can reactivate complement. Interspecies incompatibilities in complement and coagulation regulation promote persistent endothelial activation, platelet adhesion, and thrombotic microangiopathy. Furthermore, macrophages, NK cells, and neutrophils can injure the graft through Fc receptor- and complement receptor-dependent mechanisms as well as complement-independent mechanisms, including impaired inhibitory receptor–ligand signaling and missing-self recognition. Neutrophil extracellular trap formation (NETosis) amplifies endothelial injury and immunothrombosis. Ischemia–reperfusion injury and DAMP release further amplify these responses, whereas T- and B-cell responses can sustain cellular rejection and antibody production. Thus, the principal obstacle to durable survival is not a single pathway but a self-amplifying network involving antibodies, complement, cellular immunity, endothelial injury, and coagulation dysregulation (6, 13, 16, 21, 44). Accordingly, complement in xenogeneic kidney transplantation should be viewed as an early effector of hyperacute rejection and as a central component of this broader pathogenic network. Therefore, durable graft survival will require a multilayered strategy integrating antigen elimination, expression of human complement regulatory proteins, and regulation of coagulation and innate immune cells.

### 4.1 Complement regulatory proteins in genetically modified pigs

The expression of human complement regulatory proteins in donor organs is an important strategy for complement regulation in xenotransplantation. Key complement regulatory proteins include CD46/MCP, CD55/DAF, and CD59, which regulate complement activation at various stages by promoting C3b/C4b cleavage, accelerating the decay of C3/C5 convertases, and preventing MAC formation, respectively (22, 45). As these molecules regulate different stages of the complement cascade, expression of a single complement regulatory protein is unlikely to provide effective protection, highlighting the need for a strategy combining multiple human complement regulatory proteins (44, 45).

Additional complement regulatory strategies have investigated the use of molecules that regulate more upstream reactions or C4 and C3 cleavages, such as C1-INH and CD35/complement receptor 1 (CR1). C1-INH can modulate multiple inflammation- and coagulation-related pathways, including the classical and lectin pathways, and has therefore gained attention as both a pharmacological agent and a candidate molecule for transgenic expression in grafts (13). CD35/CR1 acts as a cofactor for factor I-mediated cleavage of C3b and C4b and accelerates the decay of C3/C5 convertases (22). Thus, complement regulation in genetically modified pigs can serve as a means of mitigating hyperacute rejection and a foundational technology for simultaneously regulating local complement activation, endothelial injury, and coagulation abnormalities within the graft.

### 4.2 Complement–coagulation crosstalk, endothelial injury, and thromboinflammation

In both allogeneic and xenogeneic transplantation, complement activation and coagulation abnormalities are interconnected processes that mutually amplify each other through endothelial injury (21). C5a and MAC generated during complement activation induce endothelial cell activation and promote the expression of adhesion molecules and tissue factor, enhance platelet adhesion, and induce microthrombus formation. Conversely, coagulation-related proteases such as thrombin and factor Xa can directly or indirectly activate complement components and further propagate complement reactions. This complement–coagulation crosstalk plays a central role in the pathogenesis of thrombotic microangiopathy and graft dysfunction, which are key manifestations of xenograft vascular injury (46, 47).

Recent genetically modified pigs are increasingly designed to express coagulation regulatory molecules in addition to complement regulatory proteins. Key representative molecules include human thrombomodulin, endothelial protein C receptor (EPCR), CD39, and tissue factor pathway inhibitor (TFPI) (44, 46–48). Thrombomodulin and EPCR enhance anticoagulant activity through the protein C pathway and preserve the antithrombotic properties of the endothelial surface. CD39 degrades extracellular ATP and ADP, thereby suppressing platelet activation while weakening the link between inflammation and coagulation. TFPI suppresses the tissue factor pathway and modulates excessive activation of the extrinsic coagulation pathway. When combined with complement regulatory proteins, these molecules regulate the complement–coagulation–inflammation axis in xenogeneic graft endothelium in a multilayered manner (44, 46, 47).

### 4.3 Complement and innate cellular rejection

Complement activation provides a direct mechanistic link between humoral injury and innate cellular rejection in xenotransplantation (**Figure 2**). C3a and C5a recruit and activate macrophages and neutrophils through C3aR and C5aR1, whereas iC3b deposited on porcine endothelium is recognized by CR3 and CR4 and enhances cellular adhesion and effector-cell interactions. C5a and sublytic MAC also activate graft endothelium, inducing adhesion molecules, inflammatory mediators, and tissue factor, facilitating myeloid-cell recruitment and immunothrombosis. Activated macrophages release TNF-α, IL-1β, IL-6, and reactive oxygen species, whereas neutrophils release reactive oxygen species, myeloperoxidase, neutrophil elastase, and NETs. NETs can stimulate macrophage IL-1β production, and IL-1β can enhance NETosis, leading to a self-amplifying loop that promotes endothelial injury, DAMP release, and further complement and coagulation activation (13, 16, 21, 46, 47).

**Figure 2:**
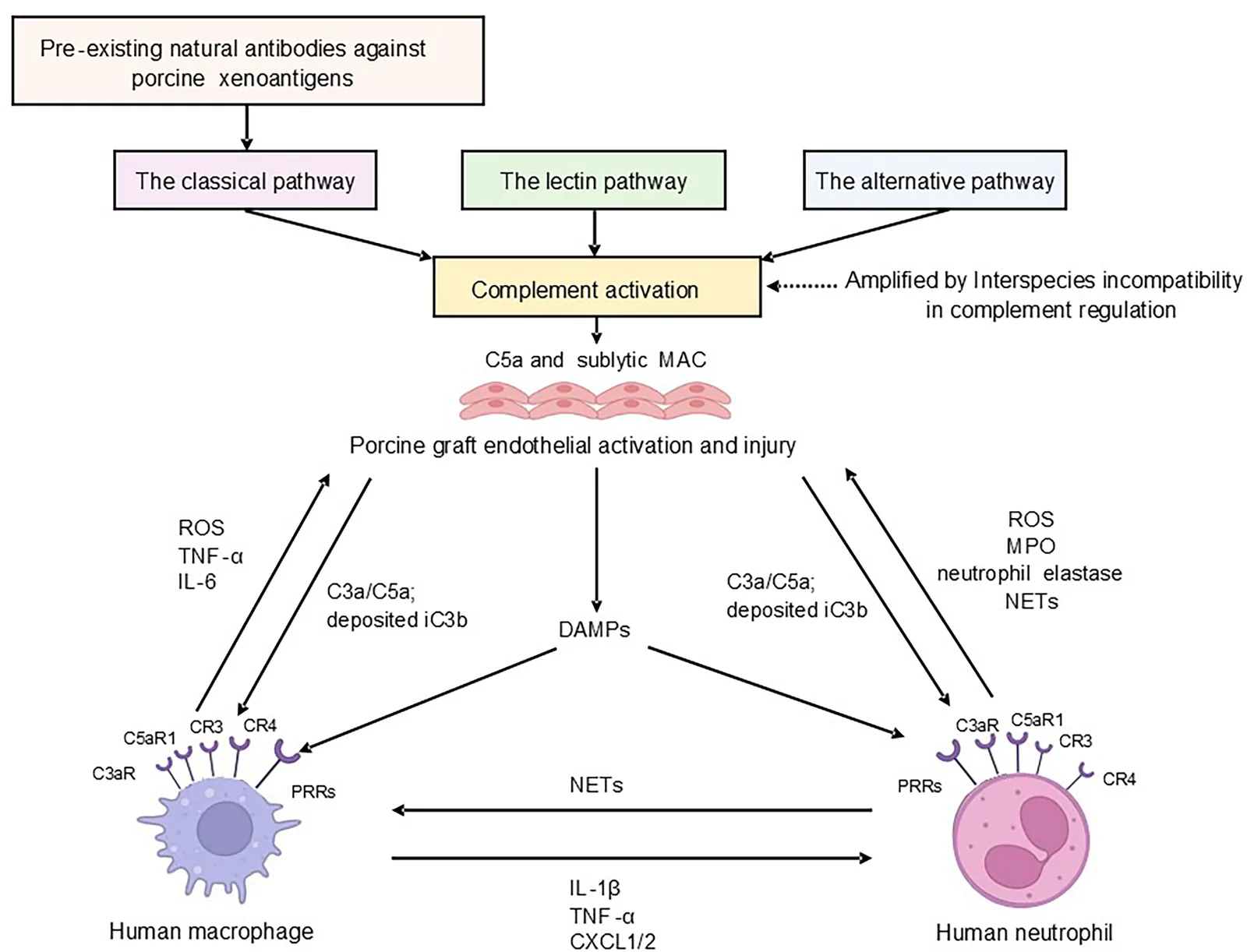
Complement-driven amplification of macrophage and neutrophil responses in kidney xenotransplantation. Pre-existing natural antibodies against porcine xenoantigens initiate complement activation primarily through the classical pathway, whereas the lectin and alternative pathways and interspecies incompatibility in complement regulation amplify and sustain complement activation. C3a and C5a recruit and activate macrophages and neutrophils through C3aR and C5aR1, whereas iC3b deposited on porcine endothelium is recognized through CR3 and CR4. C5a and sublytic MAC directly promote graft endothelial activation and injury. Activated macrophages and neutrophils release cytokines, reactive oxygen species, granule enzymes, and NETs, further damaging porcine graft endothelium. Endothelial injury induces DAMP release and PRR-mediated activation, while reciprocal interactions between macrophages and neutrophils create a self-amplifying inflammatory loop. Although these downstream complement–myeloid effector mechanisms also operate in allogeneic transplantation, they are more strongly amplified in xenotransplantation. C3aR, C3a receptor; C5aR1, C5a receptor 1; CR3/CR4, complement receptors 3/4; DAMPs, damage-associated molecular patterns; IL, interleukin; MAC, membrane attack complex; MPO, myeloperoxidase; NETs, neutrophil extracellular traps; PRRs, pattern-recognition receptors; ROS, reactive oxygen species; TNF-α, tumor necrosis factor-α.

NK-cell injury is less directly linked to complement. Xenoreactive antibodies can simultaneously activate the classical complement pathway and trigger CD16-mediated antibody-dependent cellular cytotoxicity, whereas complement-mediated endothelial activation may facilitate leukocyte recruitment. In contrast, missing-self recognition resulting from the inadequate engagement of human inhibitory receptors by porcine SLA is largely complement-independent (6, 13, 16).

Thus, complement-dependent and independent mechanisms coexist in innate cellular rejection. Despite advances in regulating complement and coagulation, innate cellular rejection remains a notable challenge in xenotransplantation. Our group has focused on innate cellular rejection mechanisms involving macrophages, NK cells, and neutrophils and identified several molecules that regulate these cells (16). CD47 binds to signal regulatory protein α (SIRPα) and transmits inhibitory signals through immunoreceptor tyrosine-based inhibitory motifs, serving as a “don’t eat me” signal. The expression of human CD47 on porcine cells suppresses phagocytosis and cytotoxicity mediated by human macrophages (49).

HLA-E and HLA-G1 also serve as key regulators of NK cells and macrophages. These nonclassical HLA class Ib molecules suppress the activation of NK cells and macrophages by engaging inhibitory receptors such as CD94/NKG2A and the immunoglobulin-like transcript family. HLA-E or HLA-G1 expression on porcine vascular endothelium may compensate for the absence of cross-species inhibitory signals and reduce innate immune cell-mediated xenogeneic cell injury (16, 50, 51).

The CD200–CD200R pathway also plays a critical role in macrophage regulation. The expression of human CD200 on porcine cells may induce inhibitory signals through CD200R and suppress macrophage-mediated phagocytosis, cytotoxicity, and inflammatory cytokine production. TIGIT has also been identified as a candidate molecule for suppressing the activation of innate immune cells, including macrophages, thereby broadening the range of immunoregulatory molecules for xenotransplantation (52, 53).

Neutrophils infiltrate xenogeneic grafts early and exacerbate endothelial injury through the generation of reactive oxygen species, release of granule enzymes, and NETosis. Notably, CD31 and CD177 have been demonstrated to play a significant role in neutrophil regulation. Human CD31 forms homophilic interactions with CD31 on neutrophils and suppresses NETosis through SHP-1-mediated inhibitory signaling. Similarly, CD177 interacts with CD31 on neutrophils and may suppress neutrophil NETosis (54, 55). Furthermore, CL-SP-D, a hybrid molecule comprising CL-P1 and surfactant protein D, transmits inhibitory signals through SIRPα and has been demonstrated to suppress human neutrophil-mediated cytotoxicity, reactive oxygen species production, and NETosis in a swine endothelial cell model. However, CL-SP-D has not yet been incorporated in genetically modified pigs, and its efficacy in organ transplantation models has not yet been established. Nonetheless, it may exert more pronounced suppressive effects than CD47 in neutrophil regulation and has therefore gained attention as a candidate molecule for innate immune regulation in xenotransplantation (56).

### 4.4 Therapeutic targeting of complement

Complement-targeted therapy in xenotransplantation can be broadly categorized into temporary pharmacological inhibition and sustained complement regulation via genetic modification of donor organs. Potential pharmacological agents include C1-INH, C3 inhibitors, C5 inhibitors, and C5a/C5aR inhibitors (57). In a case series of decedent pig-to-human kidney xenotransplantation, the anti-C5 antibody eculizumab was associated with preventing thrombotic microangiopathy (58).

The clinical use of complement inhibitors in a living recipient has been reported by Kawai et al. (15). The anti-C5 monoclonal antibody ravulizumab was administered prophylactically, and no thrombotic microangiopathy was observed in serial biopsies. During an episode of T cell-mediated rejection on day 8, the biopsy showed C3 deposition despite the absence of ABMR or thrombotic microangiopathy. Pegcetacoplan, a C3/C3b inhibitor, was therefore added to inhibit proximal complement activation. The rejection resolved following intensified immunosuppression, and the xenograft maintained life-supporting kidney function. C3 deposition persisted on the day-34 biopsy, but neither ABMR nor thrombotic microangiopathy was identified. The patient died from apparent sudden cardiac causes on day 52 with a functioning graft and no evidence of active xenograft rejection at autopsy. As this was a single case involving multiple concomitant interventions, the independent efficacy and optimal duration of ravulizumab and pegcetacoplan remain uncertain (15).

Complement inhibitors alone are unlikely to adequately regulate residual antibodies, coagulation abnormalities, and innate cellular injury involving neutrophils and macrophages (13, 16, 46, 56, 57). Accordingly, future strategies should aim to combine donor genetic modification, including the expression of human complement regulatory proteins, with pharmacological complement inhibition as needed and with approaches targeting coagulation abnormalities and innate immune cell responses (13, 16, 44, 45, 57). Molecules that directly suppress neutrophil NETosis represent promising novel adjunctive therapeutic agents that complement the effects of complement inhibition in xenogeneic graft injury (56).

## 5 Discussion

In this review, we summarized the role of the complement system in allogeneic and xenogeneic kidney transplantation. Complement has traditionally been viewed as the terminal effector of antibody-mediated tissue injury in hyperacute rejection and ABMR. However, complement should now be recognized as a central pathogenic factor that integrates antibody responses, endothelial injury, coagulation abnormalities, ischemia–reperfusion injury, and innate immune cell responses, thereby driving local inflammatory reactions within the graft.

In allogeneic kidney transplantation, activation of the classical pathway triggered by DSAs or anti-ABO blood group antibodies serves as a key mechanism underlying ABMR. Although C4d deposition is a key histological indicator of complement activation, as highlighted in ABO-incompatible kidney transplantation, C4d deposition alone does not necessarily indicate tissue injury or poor prognosis. Therefore, complement activation should be interpreted within the context of MVI, endothelial injury, complement-binding DSA, and innate immune responses involving NK cells. Furthermore, no standardized effective treatment has been established for chronic active ABMR, and defining the therapeutic window, patients, and pathological contexts in which complement should be targeted remains a critical consideration (11, 12).

In xenogeneic kidney transplantation, the importance of complement regulation is even more pronounced than in allotransplantation. The introduction of human complement regulatory proteins and knockout of major xenogeneic antigens have largely mitigated hyperacute rejection. However, residual antibodies, non-Gal antigens, lectin activation, amplification through the alternative pathways, coagulation abnormalities, and innate cellular rejection mediated by neutrophils and macrophages continue to limit long-term graft survival. Therefore, complement regulation in xenotransplantation should be considered as part of a comprehensive strategy encompassing coagulation control and innate immune cell regulation.

In future transplantation, it will be crucial to determine the optimal combination of complement inhibitors, genetically modified organs expressing complement regulatory proteins, coagulation regulatory molecules, and innate immune regulatory molecules. Nevertheless, it should be recognized that complement is essential for host defense against infection and immune homeostasis; thus, broad complement suppression carries risks of infection and immune dysregulation. Therefore, complement-targeted therapy should aim for precise regulation that considers the specific pathway, timing, localization, and pathological context of complement activation.

In conclusion, the complement system is not only an effector of graft injury but rather a therapeutically modifiable regulatory axis that links endothelial injury, coagulation abnormalities, and innate immune cell responses in both allogeneic and xenogeneic transplantation. The development of integrated immunoregulatory strategies based on complement regulation will be critical for overcoming chronic active ABMR and achieving long-term survival in xenogeneic kidney transplantation.
